# Analysis of Pregnancy-Related Attacks in Neuromyelitis Optica Spectrum Disorder: A Systematic Review and Meta-Analysis

**DOI:** 10.1001/jamanetworkopen.2022.25438

**Published:** 2022-08-04

**Authors:** Liang Wang, Manqiqige Su, Zhirui Zhou, Lei Zhou, Jingzi ZhangBao, Hongmei Tan, Wenjuan Huang, Xuechun Chang, Chuanzhen Lu, Jian Yu, Min Wang, Jiahong Lu, Chongbo Zhao, Tiansong Zhang, Chao Quan

**Affiliations:** 1 Department of Neurology and Rare Disease Center, Huashan Hospital, Shanghai Medical College, Fudan University, Shanghai, China; 2 National Center for Neurological Disorders, Shanghai, China; 3 Radiation Oncology Center, Huashan Hospital, Shanghai Medical College, Fudan University, Shanghai, China; 4 Department of Ophthalmology and Vision Science, Eye and ENT Hospital, Shanghai Medical College, Fudan University, Shanghai, China; 5 Department of Chinese Traditional Medicine, Jing’an District Hospital of Traditional Chinese Medicine, Shanghai, China

## Abstract

**Importance:**

Risk of relapse may be increased in the postpartum period of neuromyelitis optica spectrum disorder (NMOSD). Information regarding factors associated with pregnancy-related attacks is still lacking.

**Objectives:**

To identify factors associated with pregnancy-related NMOSD attacks, investigate the integrated annualized relapse rate (ARR) and Expanded Disability Status Scale (EDSS) score in each phase of pregnancy, and summarize pregnancy outcomes and complications in patients with NMOSD.

**Data Sources:**

An electronic search was performed in the MEDLINE, PubMed in-process and non-MEDLINE, EMBASE, Web of Science, and Cochrane databases using the OvidSP search platform, updated through December 30, 2021.

**Study Selection:**

All published and unpublished studies in English were considered, covering all patients with NMOSD with an informative pregnancy.

**Data Extraction and Synthesis:**

Two independent reviewers extracted the published data with a standardized procedure following MOOSE and PRISMA guidelines. The end points were calculated with the DerSimonian and Laird inverse variance (for random effects) method.

**Main Outcomes and Measures:**

The primary outcome was the rate of pregnancies with pregnancy-related NMOSD attacks, measured by risk ratios (RRs). The mean differences (MDs) in ARR and EDSS scores between each phase of pregnancy, pregnancy outcomes, and complications were defined as the secondary outcomes.

**Results:**

A total of 15 studies were analyzed, including 443 patients with NMOSD with 639 informative pregnancies. Patients receiving immunosuppressive treatment during pregnancy (RR, 0.43; 95% CI, 0.32-0.57; *P* < .001) and with older age at conception (RR, 0.67; 95% CI, 0.47-0.95; *P* = .02) had lower rates of pregnancy with pregnancy-related attacks. The increase in the ARR was highest in the first trimester after delivery compared with before pregnancy (MD, 1.28; 95% CI, 0.94-1.62; *P* < .001). The EDSS scores increased significantly both during pregnancy (MD, 0.44; 95% CI, 0.20-0.69; *P* < .001) and in the postpartum period (MD, 0.88; 95% CI, 0.51-1.26; *P* < .001) compared with before pregnancy.

**Conclusions and Relevance:**

This systematic review and meta-analysis found that receiving immunosuppressive treatment during pregnancy and older age at conception were associated with reduced risk of pregnancy-related NMOSD attacks, which mostly occurred in the first trimester of the postpartum period, although more high-quality prospective studies are needed.

## Key Points

**Question:**

What are the factors associated with pregnancy-related attacks in neuromyelitis optica spectrum disorder?

**Findings:**

This systematic review and meta-analysis of 15 studies with 443 patients found that receiving immunosuppressive treatment during pregnancy and older age at conception were associated with lower risk of pregnancy-related attacks. Annualized relapse rate was elevated especially during the first trimester after delivery while the Expanded Disability Status Scale score worsened during pregnancy and the postpartum period.

**Meaning:**

These findings suggest that receiving immunosuppressive treatment during pregnancy and older age at conception were associated with protection against pregnancy-related neuromyelitis optica spectrum disorder attacks, which mostly occurred in the first trimester of the postpartum period.

## Teaser

This systematic review and meta-analysis assesses factors associated with pregnancy-related be increased in the postpartum period of neuromyelitis attacks, annualized relapse rate, and Expanded Disability Status Scale score in each phase of pregnancy among patients with NMOSD.

## Introduction

Neuromyelitis optica spectrum disorder (NMOSD) is an autoimmune inflammatory disease of the central nervous system and predominantly involves the optic nerve and spinal cord, causing blindness and paralysis.^1^ The major pathogenic antibody of NMOSD is aquaporin-4 antibody (AQP4-Ab), while myelin oligodendrocyte glycoprotein antibody (MOG-Ab) is found in some patients with NMOSD who do not have AQP4-Ab.^2,3^

NMOSD principally affects women, many of whom develop disease activity during childbearing age. Previous studies have reported that the annualized relapse rate (ARR) among patients with NMOSD with AQP4-Ab increases especially in the first 3 months postpartum, and pregnancy outcomes include miscarriage and preeclampsia.^4,5,6^ Pregnancy-related NMOSD attacks have also been demonstrated in patients with MOG-Ab, most of which emerged in the postpartum period.^7^ Consequently, a worsening Expanded Disability Status Scale (EDSS) score has been reported during pregnancy and the postpartum period.^7,8,9^ Immunotherapy is associated with decreased risk of pregnancy-related NMOSD attacks.^10^ However, information regarding factors associated with pregnancy-related NMOSD attacks is still lacking, with only 1 systematic review without quantitative analysis and 1 meta-analysis focusing on immunosuppressive treatment retrieved.^11,12^ In this study, we aimed to identify the factors associated with pregnancy-related NMOSD attacks, investigate the integrated ARR and EDSS scores in each phase of pregnancy, and summarize pregnancy outcomes and complications in patients with NMOSD.

## Methods

We registered the systematic review protocol with the International Prospective Register of Systematic Reviews (PROSPERO).^13^ It was registered prior to conducting the review and is available to the public. This study followed the guidelines of Meta-analysis of Observational Studies in Epidemiology (MOOSE) reporting guideline and Preferred Reporting Items for Systematic Reviews and Meta-analyses (PRISMA) reporting guideline.^14,15^

### Search Strategy

Two reviewers (L.W. and M.S.) independently conducted literature searches in MEDLINE, PubMed in-process, and non-MEDLINE, EMBASE, Web of Science, and Cochrane databases through the OvidSP search platform. We combined relevant terms into a search strategy applied for these databases (eMethods in the Supplement). All published and unpublished studies in English were considered, updated through December 30, 2021, covering all patients with NMOSD with an informative pregnancy.

### Inclusion and Exclusion Criteria

Inclusion criteria were all relevant studies referred to the 2006 revised criteria for neuromyelitis optica or 2015 diagnostic criteria for NMOSD,^16,17^ from prospective cohort studies to retrospective studies. Considering that the AQP4-Ab positivity rate varied in each study, patients with MOG-Ab or seronegative status were also included. Exclusion criteria were case reports or series (ie, fewer than 10 patients), cohort studies without sufficient data, reviews, and animal studies. Two of us (L.W. and M.S.) read the studies thoroughly to evaluate appropriateness for inclusion in the systematic review. Any disagreement was arbitrated by a third investigator (Z. Z.).

### Data Extraction and Outcome Measures

We extracted accessible information regarding study year, design, and demographic and clinical characteristics of participants, as well as pregnancy outcomes and complications. Demographic and clinical features were the number of informative patients or pregnancies, AQP4-Ab serostatus, number of informative patients or pregnancies after disease onset, age at NMOSD onset, age at conception, and number of pregnancy-related NMOSD attacks. Patients who became pregnant after disease onset and had a relapse during pregnancy or within 1 year of the postpartum period were defined as having a pregnancy-related NMOSD attack. Pregnancy outcomes and complications included the numbers of term deliveries, premature deliveries, abortions (including spontaneous abortions and elective abortions), preeclampsia, and neonatal complications. Two of us (L.W. and M.S.) extracted the published data with a standardized procedure, and another reviewer (Z.Z.) rechecked the data. The corresponding authors of the included studies were contacted to acquire the unpublished data if needed. Any contradictory data were reexamined and discussed to reach consensus. In this meta-analysis, the primary outcome was the rate of pregnancies with pregnancy-related NMOSD attacks. The differences in ARR and EDSS scores between each phase of pregnancy, pregnancy outcomes, and complications were defined as the secondary outcomes.

For ARR, pregnancy phases were divided into 12 to 0 months before pregnancy, months 0 to 3 of pregnancy (T1), months 3 to 6 of pregnancy (T2), months 6 to 9 of pregnancy (T3), months 0 to 3 of the postpartum period (PP1), months 3 to 6 of the postpartum period (PP2), and months 6 to 12 of the postpartum period (PP3). For EDSS score, the pregnancy phases were divided into before pregnancy, the period during pregnancy, and months 0 to 12 of the postpartum period.

### Quality Appraisal: Risk of Bias

Using the Newcastle-Ottawa Scale specific for cohort design,^18^ 2 reviewers (L. W. and M. S.) independently evaluated the risk of bias covering selection, comparability, and outcome, with the total score ranging from 0 to 9 stars. A score of 6 or higher corresponds to low risk of bias. A funnel plot was used to assess publication bias.

### Statistical Analysis

Data analysis was conducted using Stata statistical software version 13.0 (StataCorp), while figures were constructed with Prism graphic software version 6 (GraphPad Software). The presence of heterogeneity was evaluated by the *I*^2^ test. Subgroup analysis and meta-regression analysis were conducted to explore the high heterogeneity (*I*^2^ >50% and *P* < .10). For dichotomous covariates (immunosuppressive treatment during pregnancy, age at conception [<32 vs ≥32years], AQP4-Ab, EDSS score at conception, coexisting autoimmune disease, and relapse during the year before pregnancy), risk ratios (RRs) and 95% CIs were calculated with the DerSimonian and Laird inverse variance (for random effects) method. For continuous covariates (rate of immunosuppressive treatment during pregnancy, age at conception, AQP4-Ab positivity rate, age at disease onset, and time interval from disease onset to conception), odds ratios (ORs) with 95% CIs were calculated by means of restricted maximum likelihood in meta-regression analysis. Necessary interaction analysis was also performed. For integrated ARR and EDSS scores at each phase, as well as their changing values compared with before pregnancy, mean differences (MDs) with 95% CIs were calculated with a random-effects meta-analysis model using the DerSimonian and Laird inverse variance method. The weight of each included study was calculated simultaneously to interpret the findings cautiously. The standard deviations of changing values in ARR and EDSS scores between before pregnancy and other phases were calculated using an imputed correlation coefficient.^19^ Publication bias was calculated with the Egger regression intercept test. Statistical significance was set at a 2-sided *P* < .05. Data were analyzed from January 15 to 30, 2022.

## Results

### Description of Included Studies

**Figure 1.**
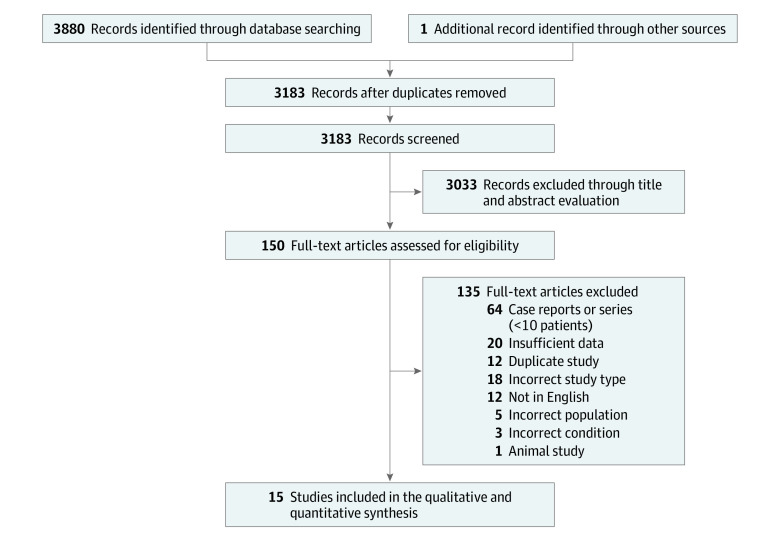
Flowchart of the Literature Search and Study Selection

A total of 3880 studies were identified in the database search, as well as 1 additional record identified through other sources, of which 3183 remained after removing duplicates. Following the first round of screening, 3033 studies were found not to be related to NMOSD and pregnancy. The remaining 150 full-text articles were sourced, and a further 135 were excluded for reasons identified in Figure 1. Ultimately, 15 studies were included in the systematic review.^4,5,6,7,8,9,12,20,21,22,23,24,25,26,27^ The demographic and clinical characteristics of the informative patients and pregnancies in NMOSD from 15 studies are summarized in Table 1. In total, 443 patients with NMOSD with 639 informative pregnancies were included, with the AQP4-Ab positivity rate ranging from 48.3% to 100%.

**Table 1.** Demographic and Clinical Characteristics of the Informative Patients and Pregnancies in Neuromyelitis Optica Spectrum Disorder From 15 Studies

Source	Type	Region	Informative patients/pregnancies, No.	AQP4-Ab positivity rate, %	Patients with pregnancies/pregnancies after disease onset, No.	Age at onset, mean (SD), y	Age at conception, mean (SD), y	Pregnancy-related attacks, No.
Bourre et al,^8^ 2012	Retrospective	France	20/25	53.3	NA/13	24.6 (7.7)	25.6 (4.3)	NA
Kim et al,^4^ 2012	Retrospective	Korea	40/54	100	26/40	25.4 (6.1)	29.9 (4.8)	34
Fragoso et al,^9^ 2013	Retrospective	Brazil	17/17	NA	17/17	26	28.2 (6.1)	NA
Nour et al,^5^ 2016	Retrospective	International	NA/27	100	NA/22	NA	28.5 (5.5)	NA
Shimizu et al,^6^ 2016	Retrospective	Japan	22/24	100	11/12	25.3 (5.8)	32.3 (5.4)	11
Huang et al,^21^ 2017	Retrospective	China	55/63	92.3	NA/30	25.6 (5.4)	25.8 (3.5)	46
Klawiter et al,^22^ 2017	Retrospective	International	NA/65	80.6	31/46	28.3 (5.7)	NA	25
Shi et al,^20^ 2017	Retrospective	China	16/22	81.3	16/22	22.9 (4.3)	28.1 (3.9)	21
Salvador et al,^23^ 2019	Retrospective	Brazil	19/30	63.2	10/21	NA	NA	33
Ashtari et al,^25^ 2020	Retrospective	Denmark	11/20	100	11/20	27.6 (10.7)	NA	15
Kim et al,^24^ 2020	Retrospective	Korea	26/33	97	26/33	25 (6.7)	32 (5.2)	17
Wang et al,^7^ 2020	Retrospective	China	110/136	75.5	60/76	23.8 (6.3)	28.1 (4.5)	69
Collongues et al,^27^ 2021	Retrospective	International	58/89	48.3	NA/67	27 (7.0)	30 (4.9)	40
Deng et al,^12^ 2021	Retrospective	China	33/34	100	22/22	27.7	27.8 (4.8)	32
Kümpfel et al,^26^ 2021	Retrospective	Germany	16/NA	NA	12/13	NA	NA	1

Abbreviations: AQP4-Ab, anti-aquaporin-4 antibody; NA, not available.

The rate of pregnancies with pregnancy-related NMOSD attacks ranged from 7.7% to 80.8% in 11 studies.^4,6,7,12,20,22,23,24,25,26,27^ However, the heterogeneity was high (*I*^2^ = 87.5%; *P* < .001) (eFigure 1 in the Supplement). Subgroup analysis and meta-regression analysis were then conducted to find the associated factors (Table 2).

**Table 2.** Factors Associated With Pregnancy-Related Neuromyelitis Optica Spectrum Disorder Attacks

Factor	No.			Effect size (95% CI)		*P* value
	Studies	Pregnancies	Events	RR	OR	
With immunosuppressive treatment during pregnancy	7	274	159	0.43 (0.32-0.57)	NA	<.001
Rate of immunosuppressive treatment during pregnancy	8	287	160	NA	0.46 (0.29-0.72)	.006
Older age at conception (≥32 y)	5	210	113	0.67 (0.47-0.95)	NA	.02
Age at conception	7	270	158	NA	0.98 (0.89-1.08)	.60
With AQP4-Ab	4	198	104	1.23 (0.83-1.82)	NA	.30
AQP4-Ab positivity rate	10	354	215	NA	1.004 (0.999-1.008)	.13
High EDSS score at conception (≥4)	4	152	89	1.36 (0.99-1.87)	NA	.06
With coexisting autoimmune disease	5	232	128	1.25 (0.99-1.57)	NA	.06
With relapse during the year before pregnancy	4	188	96	2.16 (0.73-6.41)	NA	.17
Age at disease onset	9	324	193	NA	1.00 (0.93-1.07)	.99
Time interval from disease onset to conception	6	236	134	NA	1.01 (0.87-1.18)	.81

Abbreviations: AQP4-Ab, anti-aquaporin-4 antibody; EDSS, Expanded Disability Status Scale; RR, risk ratio; OR, odds ratio.

### Factors Associated With Pregnancy-Related NMOSD Attacks

#### Immunosuppressive Treatment During Pregnancy

In the included studies, patients received immunosuppressive treatment before and during pregnancy, regardless of the duration and kind, including glatiramer acetate and interferon therapy in 1 study.^23^ There were 7 studies^6,7,12,20,23,24,27^ with 274 informative pregnancies incorporated in the subgroup analysis. The rate of pregnancy-related NMOSD attacks in the group receiving immunosuppressive treatment during pregnancy was significantly lower than that in the group without immunosuppressive treatment during pregnancy (RR, 0.43; 95% CI, 0.32-0.57; *P* < .001) (eFigure 2 in the Supplement). Eight studies^6,7,12,20,23,24,26,27^ with 287 informative pregnancies were included in the meta-regression analysis. A negative association was observed between the rate of immunosuppressive treatment during pregnancy and the rate of pregnancies with pregnancy-related NMOSD attacks (OR, 0.46; 95% CI, 0.29-0.72; *P* = .006).

#### Age at Conception

We included 5 studies^6,7,20,24,27^ with 210 informative pregnancies in the age at conception subgroup analysis. Patients were divided into the group with older age at conception (≥32 years) or younger age at conception (<32 years).^5,7^ The rate of pregnancy-related NMOSD attacks in the group with older age at conception was significantly lower than that in the group with younger age at conception (RR, 0.67; 95% CI, 0.47-0.95; *P* = .02) (eFigure 2 in the Supplement). Meta-regression analysis was also conducted in 7 studies^4,6,7,12,20,24,27^ with 270 informative pregnancies. For age at conception, the difference was not statistically significant in the rate of pregnancies with pregnancy-related NMOSD attacks (OR, 0.98; 95% CI, 0.89-1.08; *P* = .60).

#### AQP4-Ab

There were 4 studies^7,20,24,27^ with 198 informative pregnancies included in the AQP4-Ab subgroup analysis. The incidence of pregnancy-related NMOSD attacks was not different between the group with AQP4-Ab and the group without AQP4-Ab (including patients with MOG-Ab and seronegative status) (RR, 1.23; 95% CI, 0.83-1.82; *P* = .30) (eFigure 2 in the Supplement). Meta-regression analysis was also conducted with in 10 studies^4,6,7,12,20,22,23,24,25,27^ with 354 informative pregnancies. Presence of AQP4-Ab was not significantly associated with the rate of pregnancies with pregnancy-related NMOSD attacks (OR, 1.004; 95% CI, 0.999-1.008; *P* = .13).

#### EDSS Score at Conception

For EDSS score at conception, 4 studies^7,20,23,24^ with 152 informative pregnancies were included in the EDSS score subgroup analysis. Patients were dichotomized as high EDSS score (≥4) or low EDSS score (<4).^23^ There was no statistically significant difference in rate of pregnancies with pregnancy-related NMOSD attacks between the group with high EDSS score vs the group with low EDSS score (RR, 1.36; 95% CI, 0.99-1.87; *P* = .06) (eFigure 2 in the Supplement).

#### Coexisting Autoimmune Disease

We incorporated 5 studies^7,12,20,24,27^ with 232 informative pregnancies in the coexisting autoimmune disease subgroup analysis. We did not observe a statistically significant difference in rate of pregnancies with pregnancy-related NMOSD attacks in the group with coexisting autoimmune disease compared with the group without coexisting autoimmune disease (RR, 1.25; 95% CI, 0.99-1.57; *P* = .06) (eFigure 2 in the Supplement).

#### Relapse During the Year Before Pregnancy

The relapse during the year before pregnancy subgroup analysis included 4 studies^6,7,24,27^ with 188 informative pregnancies. There was no significant difference in the rate of pregnancies with pregnancy-related NMOSD attacks between the group with relapse during the year before pregnancy and the group without relapse during the year before pregnancy (RR, 2.16; 95% CI, 0.73-6.41; *P* = .17) (eFigure 2 in the Supplement).

#### Age at Disease Onset

We included 9 studies^4,6,7,12,20,22,24,25,27^ with 324 informative pregnancies in the meta-regression analysis of age at disease onset. However, age at disease onset was not observed to be associated with the rate of pregnancies with pregnancy-related NMOSD attacks (OR, 1.00; 95% CI, 0.93-1.07; *P* = .99).

#### Time Interval From Disease Onset to Conception

We incorporated 6 studies^4,6,7,20,24,27^ with 236 informative pregnancies in the meta-regression analysis of time interval from disease onset to conception. However, time interval from disease onset to conception was not found to be significantly associated with the rate of pregnancies with pregnancy-related NMOSD attacks (OR, 1.01; 95% CI, 0.87-1.18; *P* = .81).

#### Interaction Analysis

We conducted an analyses assessing potential interaction between associated factors on pregnancy-related attacks (eTable 1 in the Supplement). We did not observe any statistically significant differences.

### ARR at Each Phase

**Figure 2.**
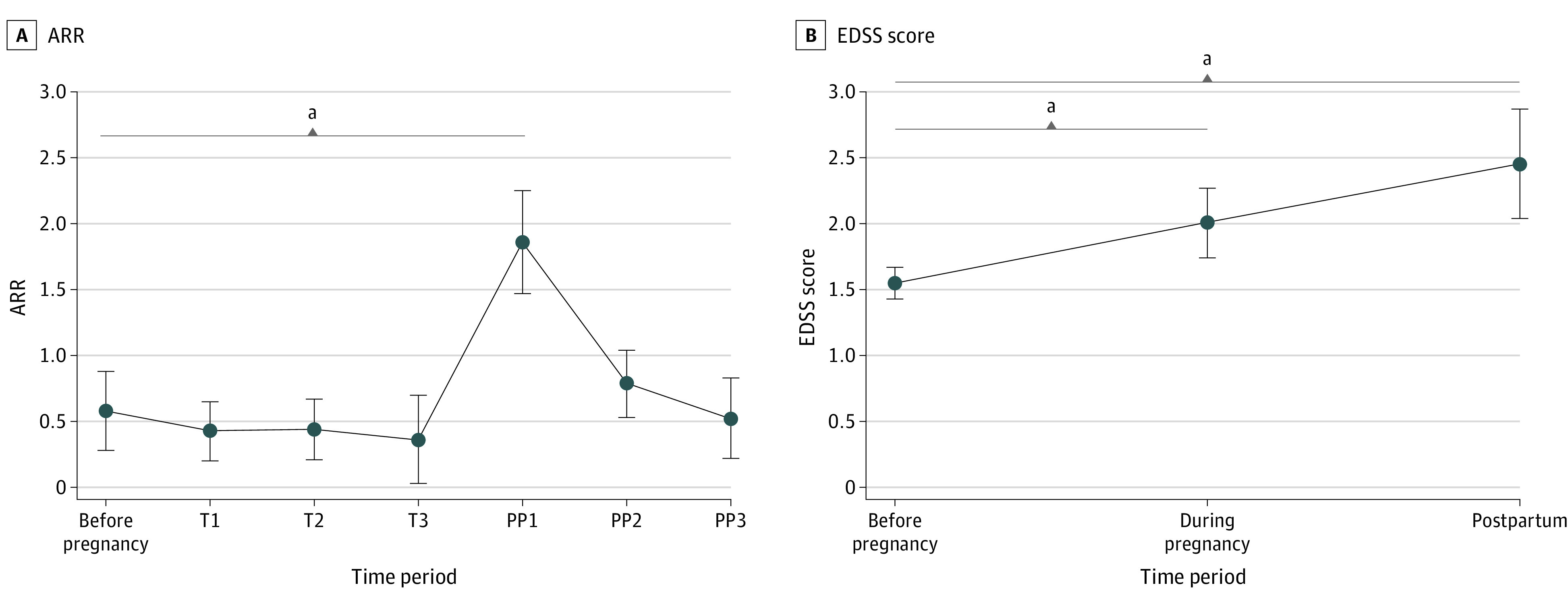
Integrated Annualized Relapse Rate (ARR) and Expanded Disability Status Scale (EDSS) Score at Each Phase Whiskers indicate 95% CIs. T1 indicates months 0 to 3 of pregnancy; T2, months 3 to 6 of pregnancy; T3, months 6 to 9 of pregnancy; PP1, months 0 to 3 of the postpartum period; PP2, months 3 to 6 of the postpartum period; PP3, months 6 to 12 of the postpartum period. Before pregnancy includes: 12 to 0 months before pregnancy and postpartum, months 0 to 12 after pregnancy. ^a^*P* < .01.

The quantitative analysis of ARR at each phase before, during, and after pregnancy included 12 studies.^4,5,6,7,8,9,12,20,21,22,23,27^ The integrated ARRs at each phase are shown in Figure 2A. The highest ARR was 1.86 (95% CI, 1.47-2.25) in PP1, while the lowest ARR was 0.36 (95% CI, 0.03-0.70) in T3. The difference in ARR was statistically significant between before pregnancy and PP1 (MD, 1.28; 95% CI, 0.94-1.62; *P* < .001) (eFigure 3 in the Supplement). However, the differences in ARR were not statistically significant between before pregnancy and the other phases of pregnancy (T1: MD, –0.17; 95% CI, –0.35 to 0.02; *P* = .08; T2: MD, –0.12; 95% CI, –0.31 to 0.07; *P* = .21; T3: MD, –0.20; 95% CI, –0.41 to 0.01; *P* = .06) or postpartum (PP2: MD, 0.27; 95% CI, –0.01 to 0.54; *P* = .06; PP3: MD, −0.03; 95% CI, –0.20 to 0.15; *P* = .78) (eFigure 3 in the Supplement). Subgroup analyses based on antibody status are presented in the eAppendix and eFigures 4 through 7 in the Supplement.

### EDSS Score at Each Phase

The EDSS score quantitative analysis included 6 studies.^7,8,9,20,21,24^ Integrated EDSS scores at each phase are presented in Figure 2B. Compared with the EDSS score before pregnancy, the EDSS scores increased significantly during pregnancy (MD, 0.44; 95% CI, 0.20-0.69; *P* < .001) and postpartum (MD, 0.88; 95% CI, 0.51-1.26; *P* < .001) (eFigure 8 in the Supplement). Subgroup analyses according to antibody status are presented in the eAppendix, eFigure 4, and eFigure 8 in the Supplement.

### Pregnancy Outcomes and Complications

Pregnancy outcomes and complications of the informative pregnancies in NMOSD from 15 studies are summarized in Table 3. Among 619 informative pregnancies, we recorded 396 term deliveries (64.0%), 30 premature deliveries (4.8%), 140 abortions (22.6%; including 50 spontaneous abortions [8.1%] and 90 elective abortions [14.5%]), and 17 patients who developed preeclampsia (2.7%). There were also 33 births (5.3%) with neonatal complications, including various health issues. Factors associated with spontaneous abortions or neonatal complications are presented in the eAppendix, eFigure 9 and eTable 2 in the Supplement.

**Table 3.** Pregnancy Outcomes and Complications of the Informative Pregnancies in Neuromyelitis Optica Spectrum Disorder From 15 Studies

Source	No.							Neonatal complications (No. affected)
	Informative pregnancies	Term deliveries	Premature deliveries	Abortions	Spontaneous abortions	Elective abortions	Preeclampsia	
Bourre et al,^8^ 2012	25	NA	NA	NA	NA	NA	0	NA
Kim et al,^4^ 2012	40	25	1	14	1	13	1	Birth defect (1)
Fragoso et al,^9^ 2013	17	15	1	1	1	0	1	NA
Nour et al,^5^ 2016	27	NA	NA	NA	NA	NA	1	Hydrocephalus (1)
Shimizu et al,^6^ 2016	24	20	1	2	0	2	1	Low birth weight (1); stillbirth (1)
Huang et al,^21^ 2017	63	42	4	17	7	10	0	Low birth weight (8); hydrocephalus (1)
Klawiter et al,^22^ 2017	46	30	4	12	10	2	3	Congenital anomalies (1); other health issues (4)
Shi et al,^20^ 2017	22	15	1	6	2	4	0	Low birth weight (1)
Salvador et al,^23^ 2019	30	20	5	5	3	2	NA	Cerebral ischemia (1)
Ashtari et al,^25^ 2020	20	14	0	6	4	2	1	Hypoxia and seizure (1); low Apgar score (1)
Kim et al,^24^ 2020	33	24	0	9	6	3	0	Low birth weight (1)
Wang et al,^7^ 2020	136	84	6	46	4	42	0	Low birth weight (4); undeveloped external ear (1); dacryocyst obstruction (1); scoliosis (1)
Collongues et al,^27^ 2021	89	76	0	13	10	3	3	NA
Deng et al,^12^ 2021	34	20	6	8	1	7	6	Low birth weight (1); splenomegaly (1); thrombocytopenia (1)
Kümpfel et al,^26^ 2021	13	11	1	1	1	0	0	NA
Total, No. (%)	619	396 (64.0)	30 (4.8)	140 (22.6)	50 (8.1)	90 (14.5)	17 (2.7)	33 (5.3)

Abbreviation: NA, not available.

### Risk of Bias Assessment

The Newcastle-Ottawa Scale for quality appraisal of the included studies is presented in eTable 3 in the Supplement. The total score ranged from 6 to 9, indicating a high quality of the included studies. Egger regression intercept test showed that the publication bias was not statistically significant (eFigure 10 in the Supplement).

## Discussion

In this systematic review and meta-analysis, we identified factors associated with pregnancy-related NMOSD attacks, investigated the integrated ARR and EDSS scores in each phase of pregnancy, and summarized pregnancy outcomes and complications. We found that receiving immunosuppressive treatment during pregnancy and older age at conception were associated with lower risk of pregnancy-related NMOSD attacks. Furthermore, compared with the prepregnancy period, ARR was elevated especially during the initial 3 months after delivery, while the EDSS score worsened during pregnancy and the postpartum period. Additionally, several pregnancy outcomes and complications were observed.

Our findings indicated that receiving immunosuppressive treatment during pregnancy was associated with lower rate of pregnancy-related NMOSD attacks. Pregnancies in patients with NMOSD should be regarded as high risk. Generally, mycophenolate mofetil, methotrexate, and mitoxantrone should be stopped before conception, and safer treatment options, like azathioprine and monoclonal antibodies, are recommended.^10^ It has been reported that using any immunosuppressive treatment, especially rituximab, before pregnancy could significantly reduce the rate of pregnancy-related NMOSD attacks.^24^ A study by Kümpfel et al^26^ reported that only 8.3% of patients with NMOSD receiving anti-CD20 therapy experienced an NMOSD relapse postpartum. Using interferon treatment could increase the relapse rate in NMOSD.^28^ Therefore, choosing appropriate immunosuppressive treatment during pregnancy to avoid pregnancy-related NMOSD attacks is of utmost importance.

We also found that age at conception (≥32 years) was associated with a lower rate of pregnancy-related NMOSD attacks, which may demonstrate the different disease activity at different ages. A study by Kunchok et al^29^ reported that older age at disease onset could lower the risk of relapse. Therefore, it is age at conception, rather than age at onset, that may be associated with pregnancy-related NMOSD attacks.

In patients with NMOSD with pregnancy-related attacks, we found elevated ARR during the first 3 months after delivery, and lower ARR during pregnancy in subgroup analyses. The results were consistent with the previous observations of multiple sclerosis (MS).^30,31^ Some studies only included patients with NMOSD with pregnancy-related attacks, which may overestimate the actual ARR during pregnancy. Even so, the statistics of ARR in most of the studies were homogenous. Discrepancies did exist for various races or ethnicities and rates of immunosuppressive treatment during pregnancy, as well as antibody status. The subgroup analysis found ARR during pregnancy was significantly lower compared with before pregnancy regardless of the antibody status. This suggests that immunological tolerance may play a role during pregnancy in patients with NMOSD, like those with MS. We also found that the EDSS score worsened during pregnancy and the postpartum period, similar to what is observed in patients with MS,^30,31^ as disability was also relapse-dependent in NMOSD. In subgroup analysis, we found EDSS score during pregnancy and the postpartum period were numerically higher in patients with AQP4-Ab than those without AQP4-Ab, which could be explained by the different pathogenesis of the disease entity.

A high rate of abortion was found in patients with NMOSD, including spontaneous and elective abortions. In this meta-analysis, the rate of spontaneous abortion was 8.1%, higher than the general population in China (2.8%) but lower than rates reported by Cohain et al (43%) and Gunnarsdottir et al (13.5%).^32,33,34^ We thought the included Chinese studies may miss spontaneous abortions that occurred early. A study by Nour et al^5^ reported that older age at conception was associated with higher odds of miscarriage in NMOSD; however the risk of miscarriage directly increases with age as well as parity. We speculate that it is age at conception that plays a major role in miscarriage rather than the disease entity. Therefore rate of spontaneous abortions was not higher in patients with NMOSD than the general population. Clinical and experimental studies have demonstrated that AQP4 is expressed in the placenta of humans and animals and have correlated AQP4-mediated placental inflammation with fetal death.^35^ Although the difference between AQP4-Ab positivity rate and the rate of pregnancies with spontaneous abortions was not statistically significant, more evidence is needed to explore the interaction.

### Limitations

This study has some limitations. The primary limitation was the retrospective nature and limited sample size of the included studies. Furthermore, some studies did not provide rigorous inclusion criteria and available comparability. Estimates calculated from imputations may affect the accuracy of conclusions to some extent.

## Conclusions

The findings of this systematic review and meta-analysis suggest that receiving immunosuppressive treatment during pregnancy and older age at conception were associated with protection against pregnancy-related NMOSD attacks. NMOSD attacks mostly occurred in the first 3 months of the postpartum period, although high-quality prospective studies are needed.

## Notes

Supplement. **eMethods. **Search Strategy for Neuromyelitis Optica **eAppendix.** ARR at Each Phase, EDSS Score at Each Phase, and Pregnancy Outcomes and Complications **eFigure 1.** Forest Plot of Rates of Pregnancy With Pregnancy-Related Attacks in Patients With NMOSD **eFigure 2.** Forest Plot of Factors Associated With Pregnancy-Related Attacks in Patients With NMOSD **eFigure 3.** Forest Plot of Differences in ARR of Patients With NMOSD Between Before Pregnancy and Other Phases **eFigure 4.** Integrated ARR and EDSS Score in Patients With NMOSD With Different Antibody Status at Each Phase **eFigure 5.** Forest Plot of Differences in ARR of Patients With AQP4-Ab Between Before Pregnancy and Other Phases **eFigure 6.** Forest Plot of Differences in ARR of Patients With MOG-Ab Between Before Pregnancy and Other Phases **eFigure 7.** Forest Plot of Differences in ARR of Patients Who Were Seronegative Between Before Pregnancy and Other Phases **eFigure 8.** Forest Plot of Differences in EDSS Score of Patients With NMOSD in Each Phase **eFigure 9.** Forest Plot of Immunosuppressive Treatment During Pregnancy on Spontaneous Abortions, or Neonatal Complications in Patients With NMOSD **eFigure 10.** Funnel Plot of Publication Bias **eTable 1.** Interaction Between Associated Factors on Pregnancy-Related NMOSD Attacks **eTable 2.** Factors Associated With Spontaneous Abortions, or Neonatal Complications **eTable 3.** The Newcastle-Ottawa Scale for Quality Appraisal of the Included Studies
