# Supplementary material for: Analysis of Pregnancy-Related Attacks in Neuromyelitis Optica Spectrum Disorder: A Systematic Review and Meta-Analysis
Source: JAMA Netw Open. 2022 Aug 4;5(8):e2225438. doi: 10.1001/jamanetworkopen.2022.25438 (PMC9353597; doi:10.1001/jamanetworkopen.2022.25438)
Supplement: Supplement. — eMethods. Search Strategy for Neuromyelitis Optica eAppendix. ARR at Each Phase, EDSS Score at Each Phase, and Pregnancy Outcomes and Complications eFigure 1. Forest Plot of Rates of Pregnancy With Pregnancy-Related Attacks in Patients With NMOSD eFigure 2. Forest Plot of Factors Associated With Pregnancy-Related Attacks in Patients With NMOSD eFigure 3. Forest Plot of Differences in ARR of Patients With NMOSD Between Before Pregnancy and Other Phases eFigure 4. Integrated ARR and EDSS Score in Patients With NMOSD With Different Antibody Status at Each Phase eFigure 5. Forest Plot of Differences in ARR of Patients With AQP4-Ab Between Before Pregnancy and Other Phases eFigure 6. Forest Plot of Differences in ARR of Patients With MOG-Ab Between Before Pregnancy and Other Phases eFigure 7. Forest Plot of Differences in ARR of Patients Who Were Seronegative Between Before Pregnancy and Other Phases eFigure 8. Forest Plot of Differences in EDSS Score of Patients With NMOSD in Each Phase eFigure 9. Forest Plot of Immunosuppressive Treatment During Pregnancy on Spontaneous Abortions, or Neonatal Complications in Patients With NMOSD eFigure 10. Funnel Plot of Publication Bias eTable 1. Interaction Between Associated Factors on Pregnancy-Related NMOSD Attacks eTable 2. Factors Associated With Spontaneous Abortions, or Neonatal Complications eTable 3. The Newcastle-Ottawa Scale for Quality Appraisal of the Included Studies [file jamanetwopen-e2225438-s001.pdf]

Supplemental Online Content

Wang L, Su M, Zhou Z, et al. Analysis of pregnancy-related attacks in neuromyelitis optica spectrum disorder: a systematic review and meta-analysis. *JAMA Netw Open*. 2022;5(8):e2225438. doi:10.1001/jamanetworkopen.2022.25438

eMethods. Search Strategy for Neuromyelitis Optica
eAppendix. ARR at Each Phase, EDSS Score at Each Phase, and Pregnancy Outcomes and Complications
eFigure 1. Forest Plot of Rates of Pregnancy With Pregnancy-Related Attacks in Patients With NMOSD
eFigure 2. Forest Plot of Factors Associated With Pregnancy-Related Attacks in Patients With NMOSD
eFigure 3. Forest Plot of Differences in ARR of Patients With NMOSD Between Before Pregnancy and Other Phases
eFigure 4. Integrated ARR and EDSS Score in Patients With NMOSD With Different Antibody Status at Each Phase
eFigure 5. Forest Plot of Differences in ARR of Patients With AQP4-Ab Between Before Pregnancy and Other Phases
eFigure 6. Forest Plot of Differences in ARR of Patients With MOG-Ab Between Before Pregnancy and Other Phases
eFigure 7. Forest Plot of Differences in ARR of Patients Who Were Seronegative Between Before Pregnancy and Other Phases
eFigure 8. Forest Plot of Differences in EDSS Score of Patients With NMOSD in Each Phase
eFigure 9. Forest Plot of Immunosuppressive Treatment During Pregnancy on Spontaneous Abortions, or Neonatal Complications in Patients With NMOSD
eFigure 10. Funnel Plot of Publication Bias
eTable 1. Interaction Between Associated Factors on Pregnancy-Related NMOSD Attacks
eTable 2. Factors Associated With Spontaneous Abortions, or Neonatal Complications
eTable 3. The Newcastle-Ottawa Scale for Quality Appraisal of the Included Studies

This supplemental material has been provided by the authors to give readers additional information about their work.

eMethods. Search Strategy for Neuromyelitis Optica

Medline, PubMed in-process and non-Medline Strategy

Exp pregnancy/ or exp fetus/ or exp pregnancy complications/ or exp infant, newborn/ or exp postpartum period/ or exp prenatal diagnosis/ or preconception care/ or (pregnan* or obstetric* or gestation*).tw. or (f?etus or f?etal).tw. or newborn.tw. or (new adj1 born).tw. or neonat*.tw. or infan*.tw. or birth*.tw. or childbirth*.tw. or (labo?r or puerper* or C?esar*).tw. or exp fetal development/ or exp fetal therapies or exp placental function tests/ or exp umbilical cord/ or exp prenatal diagnosis/ or exp fetal monitoring/ or exp perinatal care/ or exp obstetrical surgical procedures/ or exp anesthesia, obstetrical/ or exp analgesia, obstetrical/ or exp parity/ or exp apgar score/ or exp postpartum period/ or ((forcep* or vacuum or ventouse or instrument*) adj2 deliver*).tw. or (antepart* or ante-part* or prenat* or pre-nat* or perinat* or peri-nat* or peripart* or peri-part*).tw. or (postnat* or post-nat* or postpart* or post-part* or breastfe* or breast-fe* or (breast adj1 fe*)).tw. or obstetrics/

AND

Neuromyelitis Optica/ or Optic Neuritis/ or (devic or devic's or devics).mp. or neuromyelitis optica.mp. or neuromyletis optica.mp. or optic neuritis.mp. or (NMO or AQ4 or AQP4 or AQ 4 or AQP 4 or AQ-4 or AQP-4).mp. or Aquaporin 4/ or (aquaporin adj2 "4").mp.

Embase Strategy

© 2022 Wang L et al. *JAMA Network Open.*

Exp pregnancy/ or exp pregnancy disorder/ or exp pregnancy complication/ or exp pregnant women/ or exp newborn/ or (pregnan* or obstetric* or gestation*).tw. or (f?etus or f?etal).tw. or exp maternal care/ or exp puerperium/ or (newborn or (new adj1 born) or neonat* or infant*).tw. or (birth* or childbirth* or labo?r* or puerper* or c?esear* or episiotomy*).tw. or ((forcep* or vacuum or ventouse or instrument or vaginal) adj2 deliver*).tw. or (antepart* or ante-part* or prenat* or pre-nat* or antenat* or ante-nat* or perinat* or peri-nat or peripart* or peri-part*).tw. or (postnat* or post-nat* or postpart* or post-part* or lactat* or breastfe* or breast-fe*).tw. or (breast adj1 fe*).tw. or exp obstetric operation/ or exp anesthesia, obstetric/ or exp analgesia, obstetrical/ or exp parity/ or exp apgar score/ or exp postpartum period/or obstetrics/

AND

Myelooptic neuropathy/ or optic neuritis/ or (devic or devic's or devics).tw. or neuromyelitis optica.tw. or neuromyletis optica.tw. or optic neuritis.tw. or (transverse adj1 myelitis).tw. or (anti-aquaporin or

anti-NMO or anti-neuromyelitis optica or anti-AQ4).tw. or aquaporin 4 antibody/

Keyword Strategy for Web of Science and Cochrane

(pregnan* or obstetric* or gestation*) or f?etus or f?etal or newborn or neonat* or infant* or birth* or childbirth* or labo?r* or puerper* or c?esear* or episiotomy* or forcep* or vacuum or ventouse or "instrument delivery" or "vaginal delivery" or

© 2022 Wang L et al. *JAMA Network Open.*

antepart* or ante-part* or prenat* or pre-nat* or antenat* or ante-nat* or perinat* or peri-nat or peripart* or peri-part* or postnat* or post-nat* or postpart* or post-part* or lactat* or breastfe* or breast-fe*

AND

Devic or devic's or devics or neuromyelitis optica or neuromyletis optica or optic neuritis or transverse myelitis or anti-aquaporin or anti-NMO or anti-neuromyelitis optica or anti-AQ4

eAppendix. ARR at Each Phase, EDSS Score at Each Phase, and Pregnancy Outcomes and Complications

ARR at each phase

In subgroup analysis, we divided the patients with NMOSD into those with AQP4-Ab, MOG-Ab or seronegative status. There were six studies included into meta-analysis of patients with NMOSD with AQP4-Ab.[4-7, 12, 27] The integrated ARR at each phase is exhibited in eFigure 4A in the Supplement. The highest ARR was 1.64 (95% CI, 1.23-2.05) in PP1, while the lowest ARR was 0.11 (95% CI, -0.01 to 0.23) in T1. It reached the statistical significance in ARR between before pregnancy and PP1 (MD, 1.19; 95% CI, 0.64-1.74; *P* < .001), as well as before pregnancy and T1 (MD, -0.24; 95% CI, -0.42 to -0.07; *P* = .007), before pregnancy and T2 (MD, -0.19; 95% CI, -0.33 to -0.05; *P* = .009) (eFigure 5A, 5B, 5D in the Supplement). However, the differences in ARR were not significant between before pregnancy and the other phases of T3 (MD, -0.16; 95% CI, -0.36 to 0.05; *P* = .13), PP2 (MD, 0.41; 95% CI, -0.01 to 0.83; *P* = 0.06), PP3 (MD, -0.13; 95% CI, -0.31 to 0.05; *P* = .16) (eFigure 5C, 5E, 5F in the Supplement).

In meta-analysis of patients with NMOSD with MOG-Ab, there were two studies included.[7, 27] The integrated ARR at each phase is presented in eFigure 4B in the Supplement. The highest ARR was 0.63 (95% CI, -0.04 to 1.30) in PP1, while the lowest ARR was 0 (95% CI, 0-0) in T1 and T3. It reached the statistical significance in ARR between before pregnancy and T1 or T3 (MD, -0.60; 95% CI, -1.06 to -0.15; *P* = .009) (eFigure 6A, 6C in the Supplement). However, the differences in ARR were

© 2022 Wang L et al. *JAMA Network Open.*

not significant between before pregnancy and the other phases of T2 (MD, -0.28; 95% CI, -1.88 to 1.32; *P* = .73), PP1 (MD, -0.16; 95% CI, -0.79 to 0.48; *P* = .63), PP2 (MD, -0.23; 95% CI, -1.55 to 1.09; *P* = .73) and PP3 (MD, -0.31; 95% CI, -0.72 to 0.10; *P* = .13) (eFigure 6B, 6D, 6E, 6F in the Supplement).

There were two studies included into meta-analysis of patients with NMOSD with seronegative status.[7, 27] The integrated ARR at each phase is presented in eFigure 4C in the Supplement. The highest ARR was 0.83 (95% CI, -0.56 to 2.22) in PP1, while the lowest ARR was all 0 (95% CI, -0.01 to 0.01) in T1, T2 and T3. It reached the statistical significance in ARR between before pregnancy and T1, T2 or T3 (MD, -0.36; 95% CI, -0.62 to -0.10; *P* = .008) (eFigure 7A, 7B, 7C in the Supplement). However, the differences in ARR were not significant between before pregnancy and the other phases of PP1 (MD, 0.59; 95% CI, -1.03 to 2.21; *P* = .48), PP2 (MD, -0.03; 95% CI, -0.63 to 0.57; *P* = .92) and PP3 (MD, -0.22; 95% CI, -0.50 to 0.07; *P* = .13) (eFigure 7D, 7E, 7F in the Supplement).

EDSS score at each phase

In subgroup analysis, we divided the patients with NMOSD into the those with AQP4-Ab or without AQP4-Ab (including those with MOG-Ab and seronegative status). In meta-analysis of patients with NMOSD with AQP4-Ab, there were two studies included.[7, 20] The integrated EDSS score at each phase is presented in eFigure 4D in the Supplement. Compared to the EDSS score in before pregnancy, the increase in EDSS score of during pregnancy did not reach statistical significance (MD, 0.38; 95% CI, -0.08 to 0.84; *P* = .10) while that of postpartum was statistically significant

© 2022 Wang L et al. *JAMA Network Open.*

(MD, 0.60; 95% CI, 0.20-1.01; P = .004) (eFigure 8C, 8D in the Supplement).

There were two studies included into meta-analysis of patients with NMOSD without AQP4-Ab.[7, 20] The integrated EDSS score at each phase is presented in eFigure 4E in the Supplement. Compared to the EDSS score in before pregnancy, the increase in EDSS scores of during pregnancy was not statistically significant (MD, 0.18; 95% CI, -0.33 to 0.70; P = .48) while that of postpartum reached statistical significance (MD, 0.44; 95% CI, 0.03-0.86; P = .04) (eFigure 8E, 8F in the Supplement).

Pregnancy outcomes and complications

Factors associated with spontaneous abortions, or neonatal complications are presented in eTable 2 in the Supplement. It was not statistically significant in the rate of pregnancies with spontaneous abortions (RR, 1.78; 95% CI, 0.80-3.96; P = .16) or neonatal complications (RR, 1.98; 95% CI, 0.66-5.89; P = .22) between the two groups receiving or without immunosuppressive treatment during pregnancy (eFigure 9A, 9B in the Supplement). In meta-regression analysis, the rate of pregnancies with spontaneous abortions did not reach statistical significance using the rate of immunosuppressive treatment during pregnancy (OR, 1.11; 95% CI, 0.85-1.45; P = .38), age at conception (OR, 1.01; 95% CI, 0.98-1.04; P = .55), or AQP4-Ab positivity rate (OR, 1.00; 95% CI, 1.00-1.00; P = .57). The rate of pregnancies with neonatal complications did not reach statistical significance using the rate of immunosuppressive treatment during pregnancy (OR, 0.96; 95% CI, 0.76-1.21; P = .62), age at conception (OR, 0.99; 95% CI, 0.97-1.00; P = .09), or AQP4-Ab positivity rate (OR, 1.00; 95% CI,

© 2022 Wang L et al. *JAMA Network Open.*

1.00-1.00; *P* = .82).

eFigure 1. Forest Plot of Rates of Pregnancy With Pregnancy-Related Attacks in Patients With NMOSD

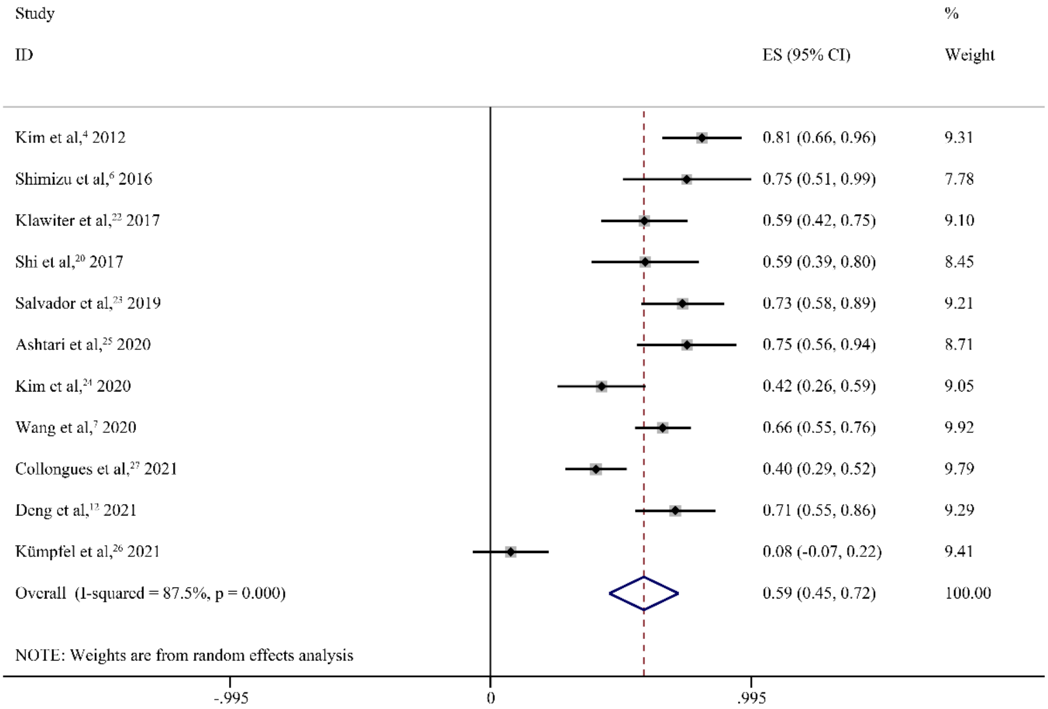

The shaded boxes around the effect sizes represent the weight of each study and whiskers indicate 95% CIs.

© 2022 Wang L et al. *JAMA Network Open.*

eFigure 2. Forest Plot of Factors Associated With Pregnancy-Related Attacks in Patients With NMOSD

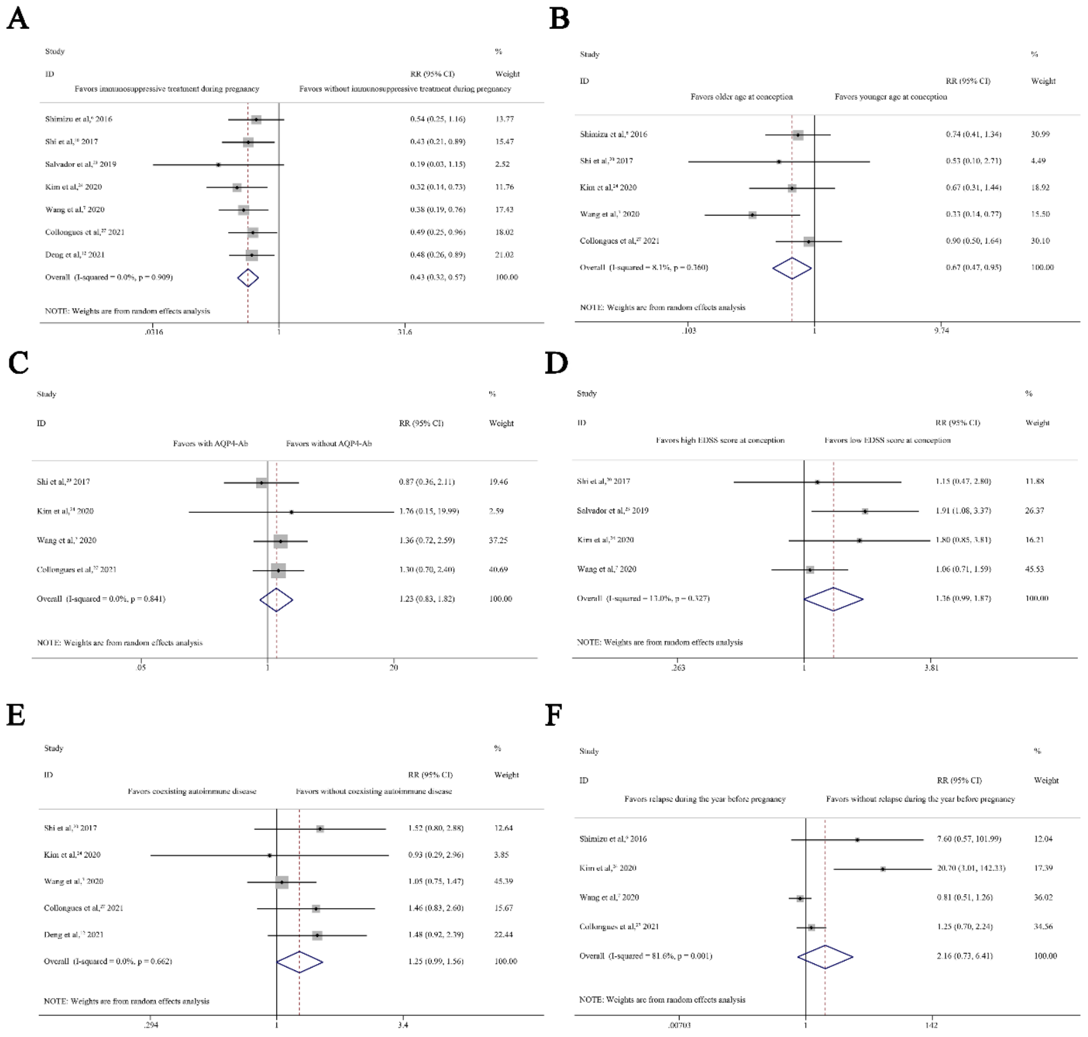

The shaded boxes around the effect sizes represent the weight of each study and whiskers indicate 95% CIs. A. Immunosuppressive treatment during pregnancy. B. Age at conception. C. AQP4-Ab. D. EDSS score at conception. E. Coexisting autoimmune disease. F. Relapse during the year before pregnancy. G. Age at disease onset. H. Time interval from disease onset to conception. NMOSD: neuromyelitis optica spectrum disorder; EDSS: Expanded Disability Status Scale; AQP4-Ab: anti-aquaporin-4 antibody.

© 2022 Wang L et al. *JAMA Network Open.*

eFigure 3. Forest Plot of Differences in ARR of Patients With NMOSD Between Before Pregnancy and Other Phases

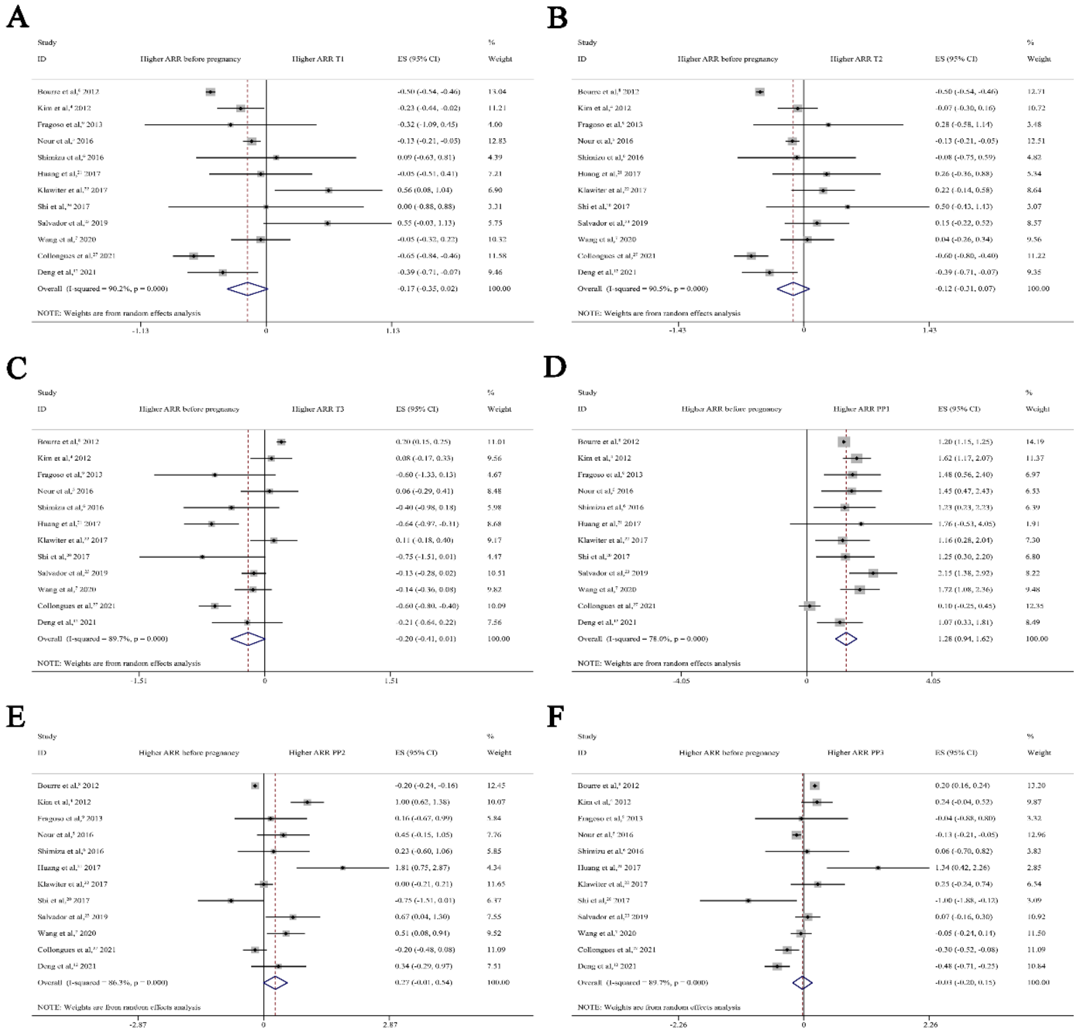

The shaded boxes around the effect sizes represent the weight of each study and whiskers indicate 95% CIs. A. Difference in ARR between before pregnancy and T1. B. Difference in ARR between before pregnancy and T2. C. Difference in ARR between before pregnancy and T3. D. Difference in ARR between before pregnancy and T1. E. Difference in ARR between before pregnancy and T2. F. Difference in ARR between before pregnancy and PP3. ARR: annualized relapse rate; T1 indicates months 0 to 3 of pregnancy; T2, months 3 to 6 of pregnancy; T3, months 6 to 9 of pregnancy; PP1, months 0 to 3 of the postpartum period; PP2, months 3 to 6 of the postpartum period; PP3, months 6-12 of the postpartum period. Before pregnancy includes: 12 to 0 months before pregnancy.

© 2022 Wang L et al. *JAMA Network Open.*

eFigure 4. Integrated ARR and EDSS Score in Patients With NMOSD With Different Antibody Status at Each Phase

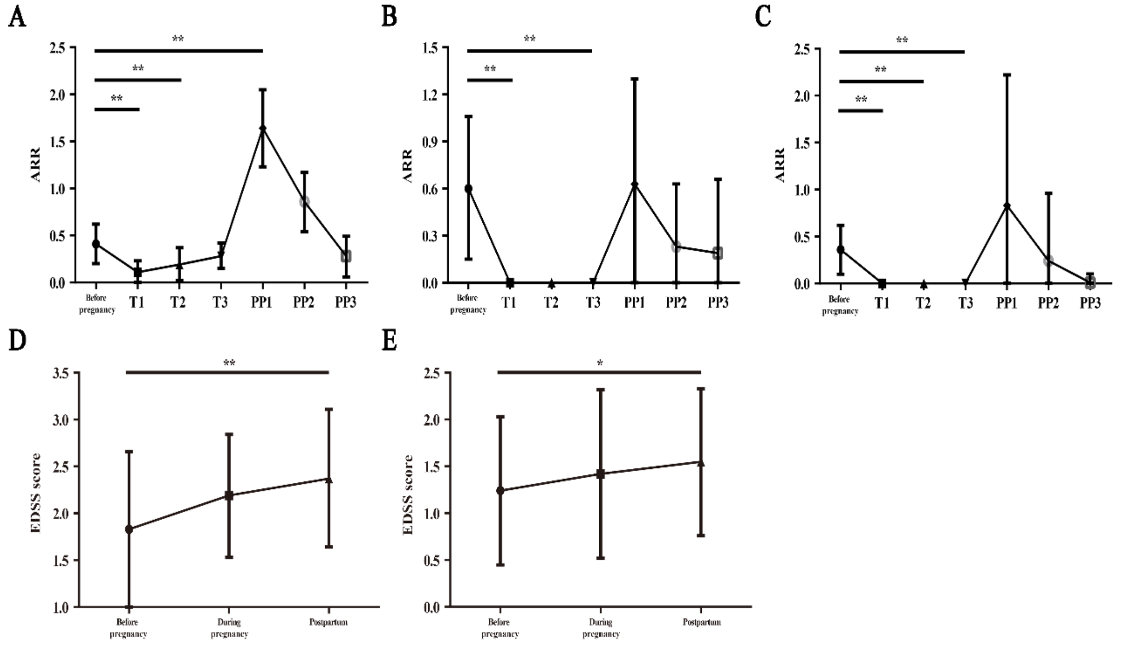

Whiskers indicate 95% CIs. A. ARR in patients with NMOSD with AQP4-Ab. B. ARR in patients with NMOSD with MOG-Ab; C. ARR in patients with NMOSD with seronegative status; D. EDSS score in patients with NMOSD with AQP4-Ab. E. EDSS score in patients with NMOSD without AQP4-Ab. ARR: annualized relapse rate; EDSS: Expanded Disability Status Scale; T1 indicates months 0 to 3 of pregnancy; T2, months 3 to 6 of pregnancy; T3, months 6 to 9 of pregnancy; PP1, months 0 to 3 of the postpartum period; PP2, months 3 to 6 of the postpartum period; PP3, months 6 to 12 of the postpartum period. Before pregnancy includes: 12 to 0 months before pregnancy and postpartum, months 0 to 12 after pregnancy. *$P < .05$; **$P < .01$.

© 2022 Wang L et al. *JAMA Network Open.*

eFigure 5. Forest Plot of Differences in ARR of Patients With AQP4-Ab Between Before Pregnancy and Other Phases

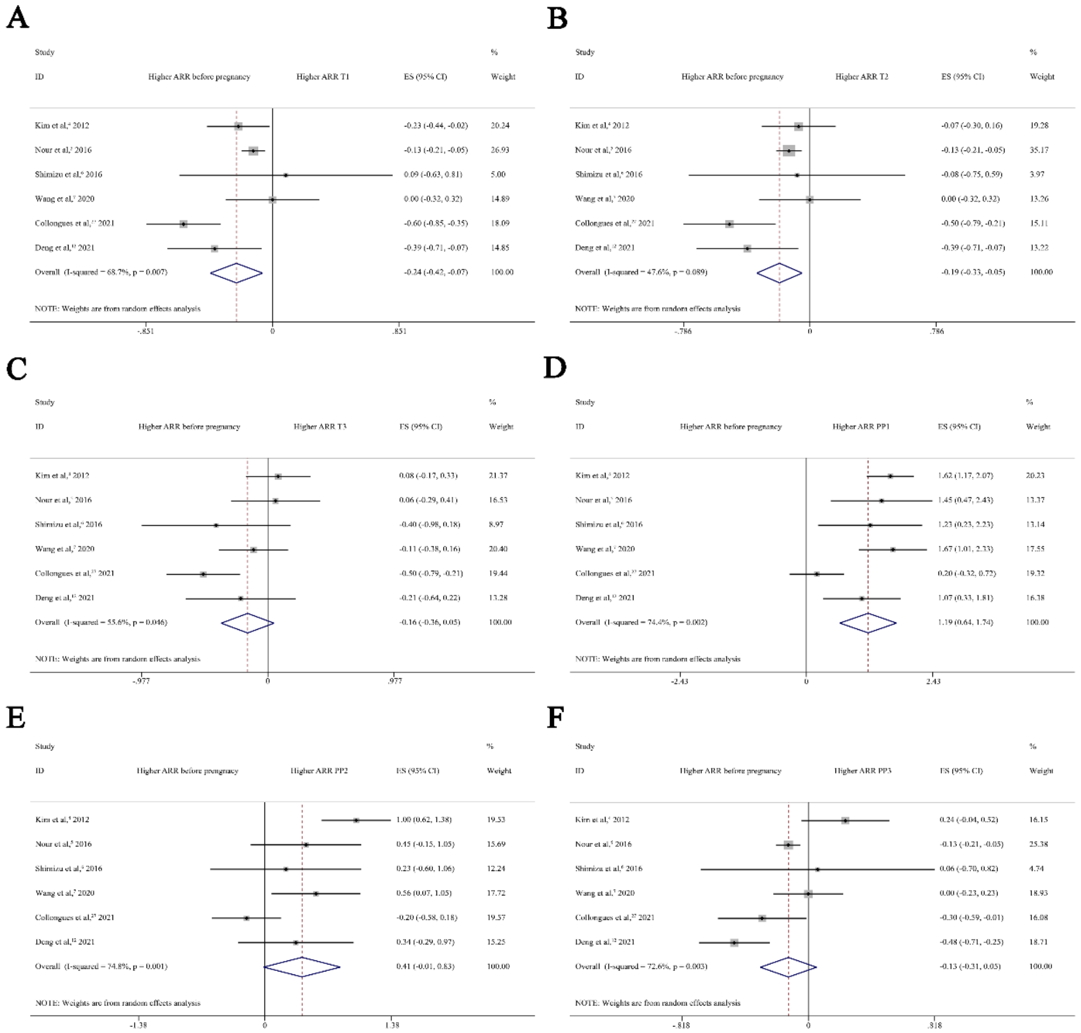

The shaded boxes around the effect sizes represent the weight of each study and whiskers indicate 95% CIs. A. Difference in ARR between before pregnancy and T1. B. Difference in ARR between before pregnancy and T2. C. Difference in ARR between before pregnancy and T3. D. Difference in ARR between before pregnancy and PP1. E. Difference in ARR between before pregnancy and PP2. F. Difference in ARR between before pregnancy and PP3. ARR, annualized relapse rate; T1 indicates months 0 to 3 of pregnancy; T2, months 3 to 6 of pregnancy; T3, months 6 to 9 of pregnancy; PP1, months 0 to 3 of the postpartum period; PP2, months 3 to 6 of the postpartum period; PP3, months 6 to 12 of the postpartum period. Before pregnancy includes: 12 to 0 months before pregnancy.

© 2022 Wang L et al. *JAMA Network Open.*

eFigure 6. Forest Plot of Differences in ARR of Patients With MOG-Ab Between Before Pregnancy and Other Phases

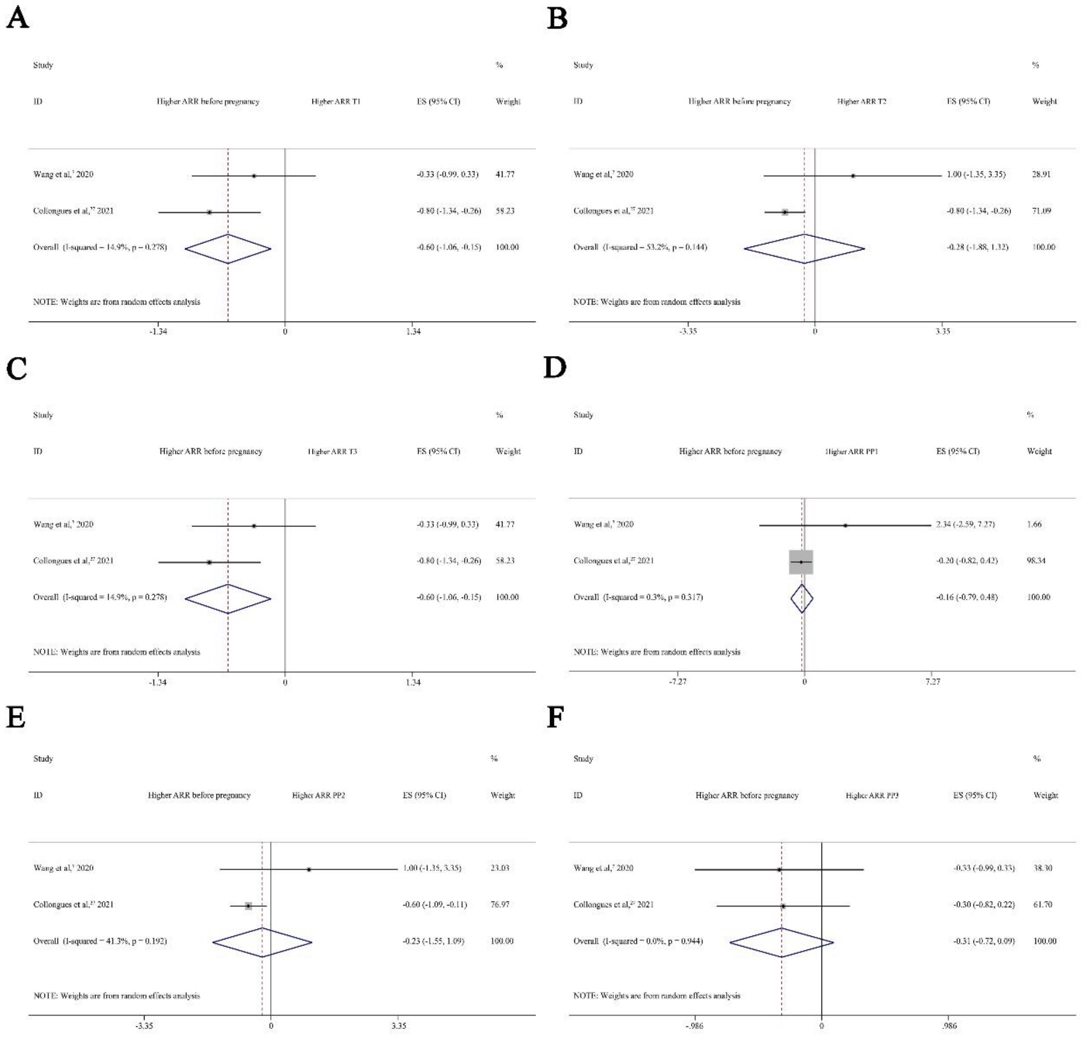

The shaded boxes around the effect sizes represent the weight of each study and whiskers indicate 95% CIs. A. Difference in ARR between before pregnancy and T1. B. Difference in ARR between before pregnancy and T2. C. Difference in ARR between before pregnancy and T3. D. Difference in ARR between before pregnancy and PP1. E. Difference in ARR between before pregnancy and PP2. F. Difference in ARR between before pregnancy and PP3. ARR: annualized relapse rate; T1 indicates months 0 to 3 of pregnancy; T2, months 3 to 6 of pregnancy; T3, months 6 to 9 of pregnancy; PP1, months 0 to 3 of the postpartum period; PP2, months 3 to 6 of the postpartum period; PP3, months 6-12 of the postpartum period. Before pregnancy includes: 12 to 0 months before pregnancy.

© 2022 Wang L et al. *JAMA Network Open.*

eFigure 7. Forest Plot of Differences in ARR of Patients Who Were Seronegative Between Before Pregnancy and Other Phases

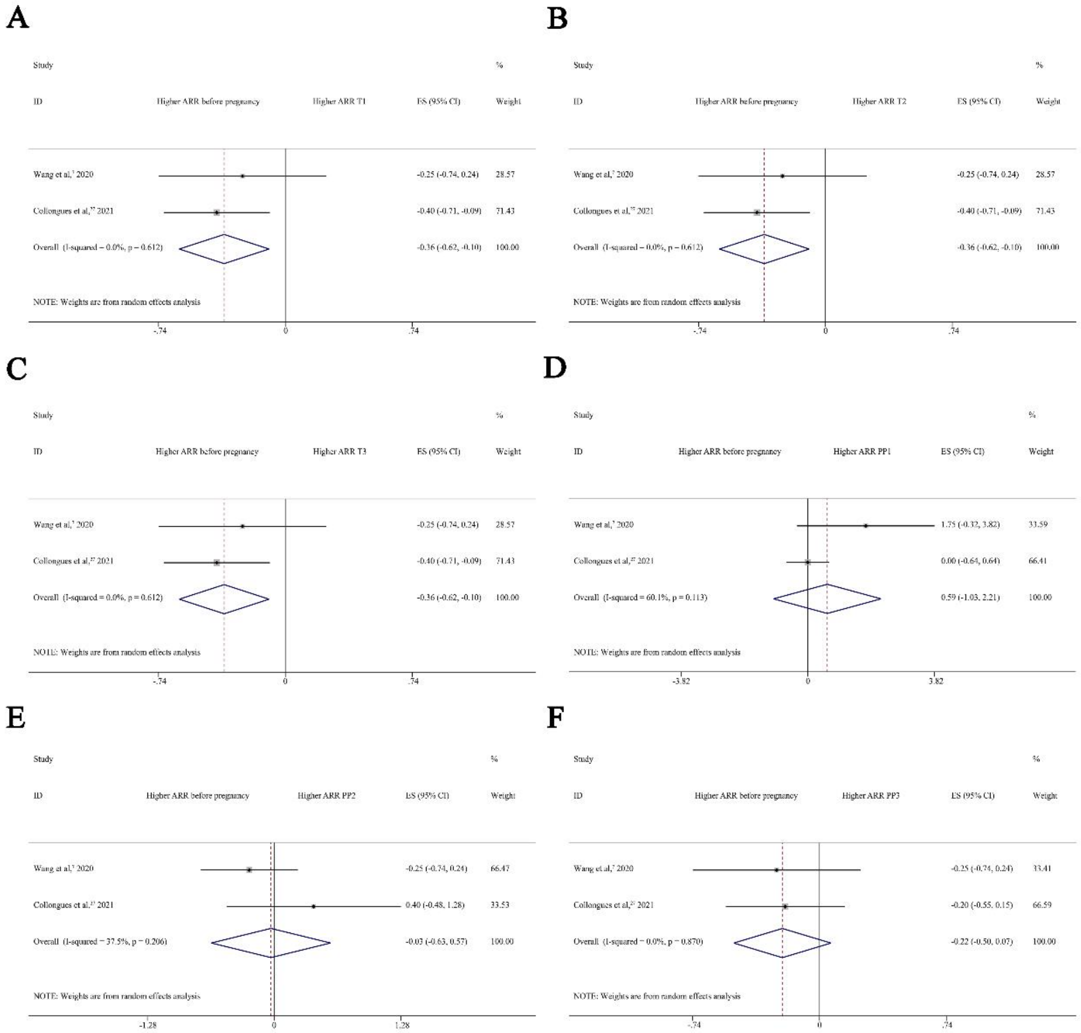

The shaded boxes around the effect sizes represent the weight of each study and whiskers indicate 95% CIs. A. Difference in ARR between before pregnancy and T1. B. Difference in ARR between before pregnancy and T2. C. Difference in ARR between before pregnancy and T3. D. Difference in ARR between before pregnancy and PP1. E. Difference in ARR between before pregnancy and PP2. F. Difference in ARR between before pregnancy and PP3. ARR: annualized relapse rate; T1 indicates months 0 to 3 of pregnancy; T2, months 3 to 6 of pregnancy; T3: months 6 to 9 of pregnancy; PP1: months 0 to 3 of the postpartum period; PP2: months 3 to 6 of the postpartum period; PP3, months 6 to 12 of the postpartum period. Before pregnancy includes: 12 to 0 months before pregnancy.

© 2022 Wang L et al. *JAMA Network Open.*

eFigure 8. Forest Plot of Differences in EDSS Score of Patients With NMOSD in Each Phase

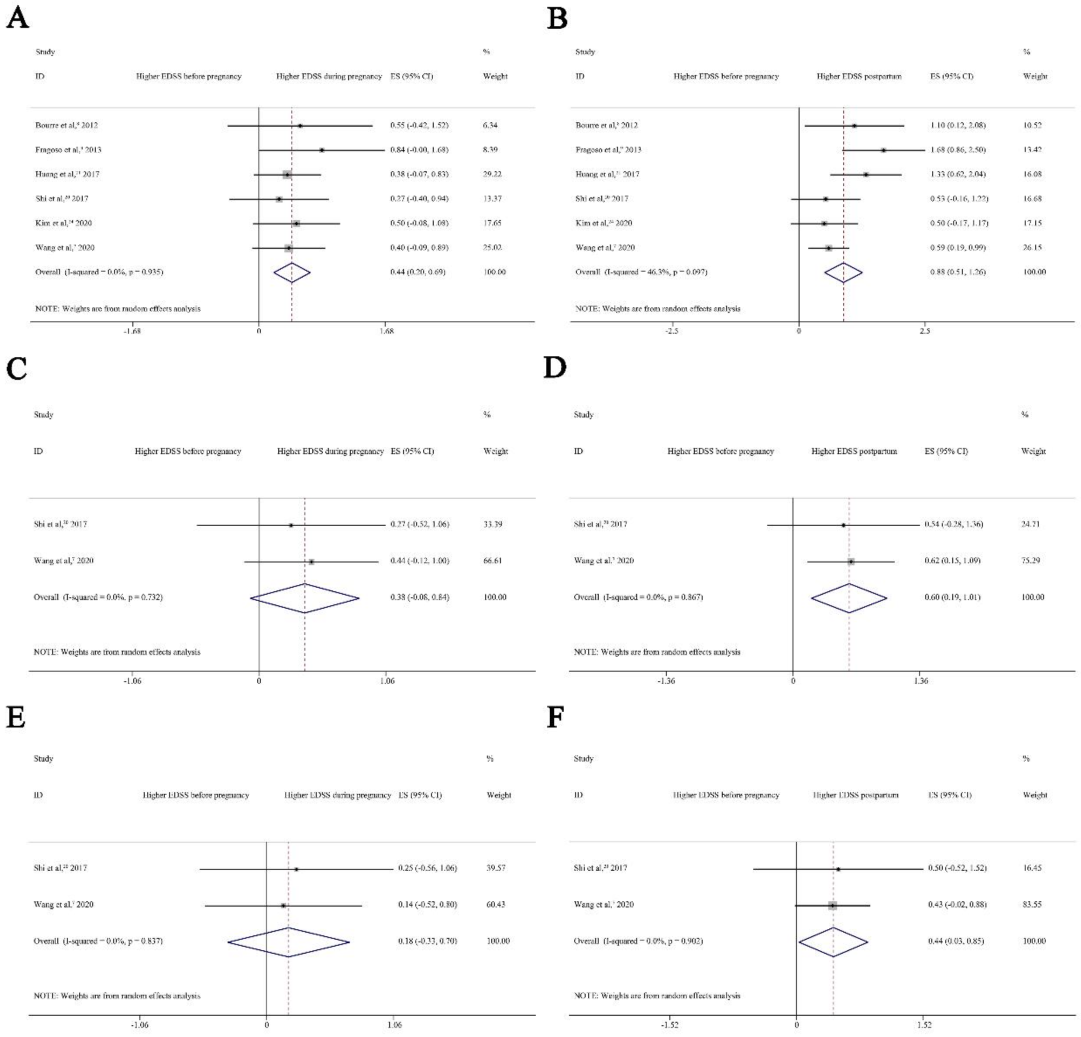

The shaded boxes around the effect sizes represent the weight of each study and whiskers indicate 95% CIs. A. Difference in EDSS score between before pregnancy and during pregnancy in all patients with NMOSD. B. Difference in EDSS score between before pregnancy and postpartum in all patients with NMOSD. C. Difference in EDSS score between before pregnancy and during pregnancy in patients with NMOSD with AQP4-Ab. D. Difference in EDSS score between before pregnancy and postpartum in patients with NMOSD with AQP4-Ab. E. Difference in EDSS score between before pregnancy and during pregnancy in patients with NMOSD without AQP4-Ab. F. Difference in EDSS score between before pregnancy and postpartum in patients with NMOSD without AQP4-Ab. EDSS: Expanded Disability Status Scale; Before

© 2022 Wang L et al. *JAMA Network Open.*

pregnancy includes: 12 to 0 months before pregnancy and postpartum, months 0 to 12 after pregnancy.

© 2022 Wang L et al. *JAMA Network Open.*

eFigure 9. Forest Plot of Immunosuppressive Treatment During Pregnancy on Spontaneous Abortions, or Neonatal Complications in Patients With NMOSD

A

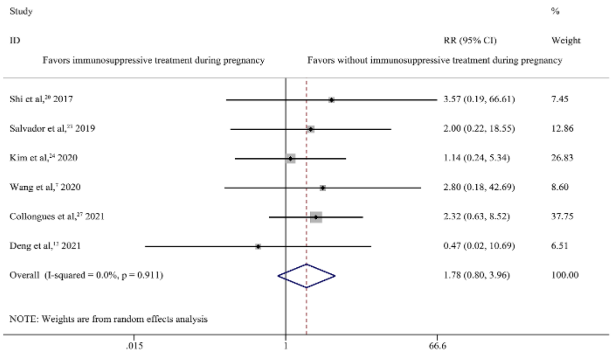

B

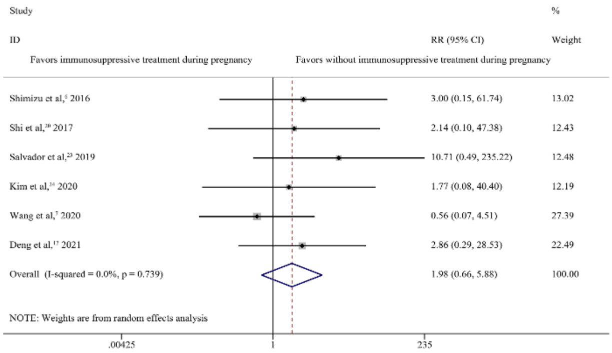

The shaded boxes around the effect sizes represent the weight of each study and whiskers indicate 95% CIs. A. Spontaneous abortions. B. Neonatal complications.

© 2022 Wang L et al. *JAMA Network Open.*

eFigure 10. Funnel Plot of Publication Bias

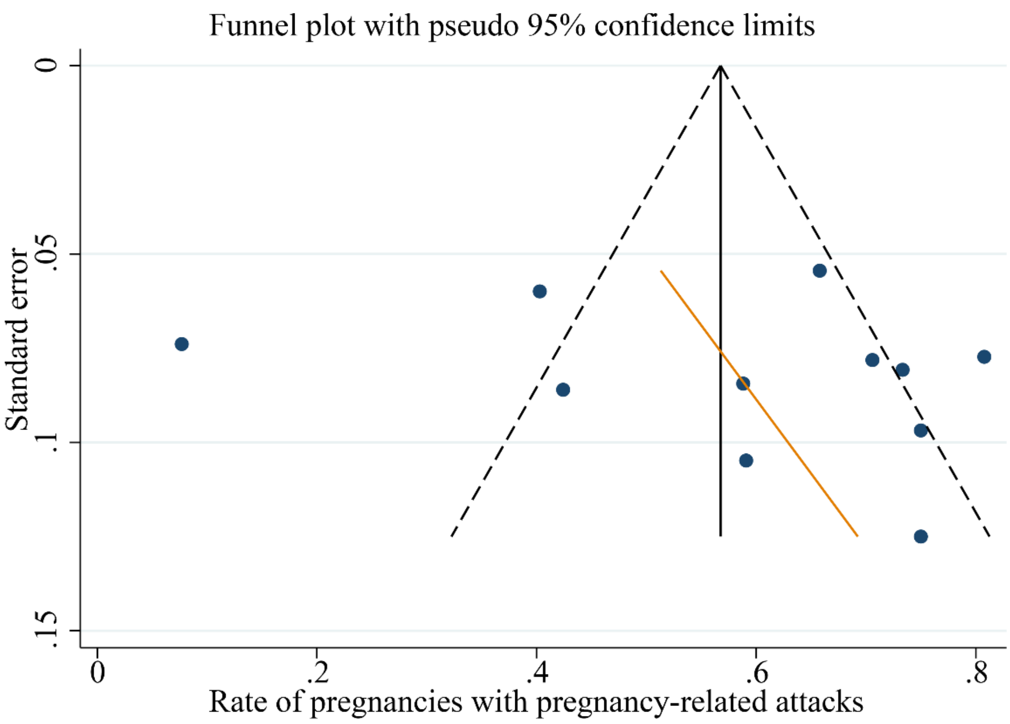

The distribution of the points indicates publication bias of the included studies.

© 2022 Wang L et al. *JAMA Network Open.*

**eTable 1.** Interaction Between Associated Factors on Pregnancy-Related Neuromyelitis Optica Spectrum Disorder Attacks				
Associated Factors	No. of studies	No. of pregnancies	No. of events	*P* value
Rate of immunosuppressive treatment during pregnancy & Age at conception	6	244	137	.81
Rate of immunosuppressive treatment during pregnancy & Age at disease onset	6	244	137	.55
Rate of immunosuppressive treatment during pregnancy & ARR before pregnancy	6	241	145	.36
ARR before pregnancy & Age at conception	6	237	144	.13
ARR before pregnancy & Age at disease onset	7	271	164	.99
Age at conception & Age at disease onset	7	270	158	.48
Abbreviations: ARR, annualized relapse rate.				

**eTable 2.** Factors Associated With Spontaneous Abortions, or Neonatal Complications						
Associated factors	No. of studies	No. of pregnancies	No. of events	Effect size	95% CI	*P* value
Immunosuppressive treatment during pregnancy†	7	274	23	RR: 1.78	0.80-3.96	.16
Immunosuppressive treatment during pregnancy‡	6	207	13	RR: 1.98	0.66-5.89	.22
Rate of immunosuppressive treatment during pregnancy†	8	287	24	OR: 1.11	0.85-1.45	.38
Rate of immunosuppressive treatment during pregnancy‡	6	207	13	OR: 0.96	0.76-1.21	.62
Age at conception†	9	364	29	OR: 1.01	0.98-1.04	.55
Age at conception‡	8	307	23	OR: 0.99	0.97-1.00	.09
AQP4-Ab positivity rate†	11	443	45	OR: 1.00	1.00-1.00	.57
AQP4-Ab positivity rate‡	11	403	31	OR: 1.00	1.00-1.00	.82
Abbreviations: AQP4-Ab, anti-aquaporin-4 antibody; RR, risk ratio; OR, odds ratio; †Rate of pregnancies with spontaneous abortions; ‡Rate of pregnancies with neonatal complications.						

© 2022 Wang L et al. *JAMA Network Open.*

eTable 3. The Newcastle-Ottawa Scale for Quality Appraisal of the Included Studies

Study	Selection				Comparability	Outcome			Total score
	Representativeness of the exposed cohort	Selection of the non-exposed cohort	Ascertainment of exposure	Demonstration that outcome of interest was not present at start of study	Comparability of cohorts on the basis of the design or analysis	Assessment of outcome	Long enough follow-up for outcomes to occur	Adequacy of follow-up of cohorts	
Bourre et al,[8] 2012	☆	☆	☆	☆	☆	☆	☆	☆	8
Kim et al,[4] 2012	☆	☆	☆	☆	☆☆	☆	☆	☆	9
Fragoso et al,[9] 2013	☆	☆	☆	☆		☆	☆	☆	7
Nour et al,[5] 2016	☆	☆	☆	☆	☆	☆	☆	☆	8
Shimizu et al,[6] 2016	☆	☆	☆	☆	☆☆	☆	☆	☆	9
Huang et al,[21] 2017	☆	☆	☆	☆	☆☆	☆	☆	☆	9
Klawiter et al,[22] 2017	☆	☆		☆		☆	☆	☆	6
Shi et al,[20] 2017	☆	☆	☆	☆	☆	☆	☆	☆	8
Salvador et al,[23] 2019	☆	☆	☆	☆	☆☆	☆	☆	☆	9
Ashtari et al,[25] 2020	☆	☆	☆	☆		☆	☆	☆	7
Kim et al,[24] 2020	☆	☆	☆	☆	☆☆	☆	☆	☆	9
Wang et al,[7] 2020	☆	☆	☆	☆	☆☆	☆	☆	☆	9
Collongues et al,[27] 2021	☆	☆	☆	☆	☆☆	☆	☆	☆	9
Deng et al,[12] 2021	☆	☆	☆	☆	☆☆	☆	☆	☆	9
Kümpfel et al,[26] 2021		☆	☆	☆	☆	☆	☆	☆	7

The total score ranges from 0 to 9 stars. A score of 6 or higher corresponds to low risk of bias.

© 2022 Wang L et al. *JAMA Network Open.*
